# Assessment of cardiovascular magnetic resonance aortic stiffness in patients with increased cardiovascular risk: role of traditional risk factors and lung hyperinflation

**DOI:** 10.1186/1532-429X-17-S1-P398

**Published:** 2015-02-03

**Authors:** Mohammed Y Khanji, Ian S Stone, Armida Balawon, Wai-Yee James, Redha Boubertakh, Neil C Barnes, Steffen E Petersen

**Affiliations:** 1 Centre for Advanced Cardiovascular Imaging and Research, William Harvey Research Institute, Queen Mary University London, London, UK; 2 Cardiology, Barts Health NHS Trust, London, UK; 3 Respiratory Medicine, Barts Health NHS Trust, London, UK; 4 Global Respiratory Franchise, GSK, Stokley Park, UK

## Background

Cardiovascular disease (CVD) accounts for up to 50% of all deaths in patients with Chronic Obstructive Pulmonary Disease (COPD). Increased arterial stiffness has been shown to predict cardiovascular events beyond the current traditional risk factors.

We sought to assess the relationship of aortic stiffness in patients with elevated global cardiovascular risk against those with COPD and lung hyperinflation.

## Methods

We assessed 82 patients with elevated cardiovascular risk as estimated by the Qrisk prediction model used in the United Kingdom; 46 were in the HAPPY London study and had normal lung function, 36 had COPD and lung hyperinflation from the DEFLATA study. CMR cine SSFP cross-section images were obtained perpendicular to the ascending thoracic (TAA), descending thoracic (TDA) and abdominal aorta (AbA). Central aortic pulse pressure was measured at the time of the scan (Vicorder device). Strain was derived as relative aortic area change. Distensibility was derived by normalizing strain to central pulse pressure.

## Results

**Figure 1:**
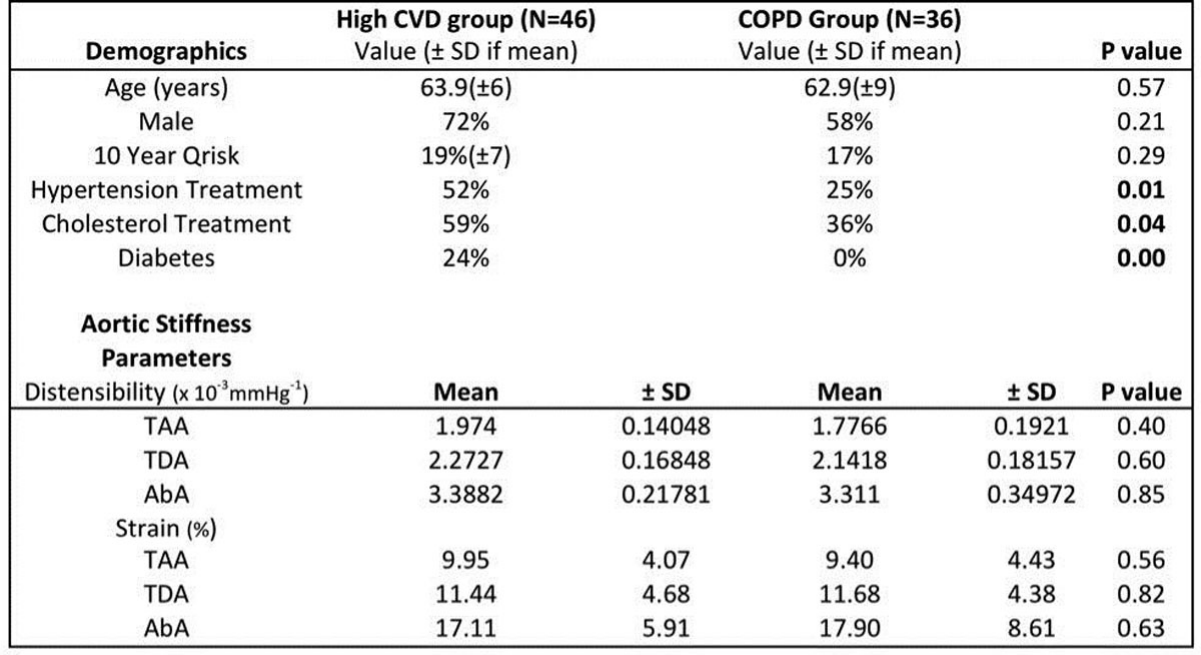
Demographics of the two groups and aortic stiffness parameters assessed.

**Figure 2:**
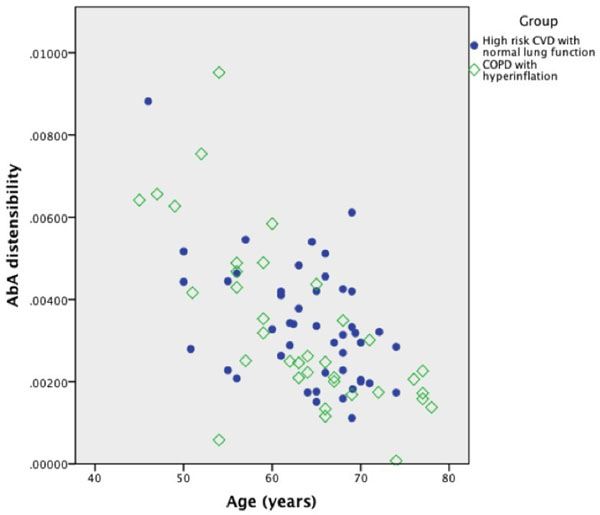
Correlation between age and AbA distensibility in the high risk high CVD & COPD group.

The mean age in the two groups was 63 years. There was no difference in the age, sex, and global cardiovascular risk scores between the groups. There was no significant difference in the aortic strain or distensibility measures between them (see Figure 1) despite the fact that diabetes, hypertension and hypercholesterolaemia was significantly lower in the COPD group. In linear regression analysis age was the only factor to significantly predict aortic strain independent of the other cardiovascular risk factors in both the groups. The strongest correlation with age was for AbA distensibility (Pearson's correlation coefficient -0.614, p<0.000, Figure 2).

## Conclusions

Aortic distensibility and strain did not differ between high-risk CVD patients with normal lung function and COPD patients with hyperinflation despite significantly lower CVD risk factors in the COPD group, suggesting that COPD may contribute to stiffness over and above traditional risk factors. Age appears to be the strongest predictor of aortic strain and distensibility. Further studies on the physiological impact of lung hyperinflation on aortic function and the relative contribution of risk factors is warranted to best guide CVD prevention in these high-risk groups.

## Funding

MYK has received funding from Barts Charity and ISS has received fundings from GSK.

